# Microwave-Assisted Lignin Extraction—Utilizing Deep Eutectic Solvents to Their Full Potential

**DOI:** 10.3390/polym14204319

**Published:** 2022-10-14

**Authors:** Alina Meindl, Alexander Petutschnigg, Thomas Schnabel

**Affiliations:** 1 Forest Products Technology and Timber Constructions Department, Salzburg University of Applied Sciences, Markt 136a, 5431 Kuchl, Austria; 2 Salzburg Center for Smart Materials, Jakob-Haringer Straße 2a, 5020 Salzburg, Austria; 3 Department of Material Sciences and Process Engineering, University of Natural Resources and Life Sciences (BOKU), Konrad Lorenz-Straße 24, 3340 Tulln, Austria; 4 Faculty of Furniture Design and Wood Engineering, Transilvania University of Brasov, B-dul. Eroilor nr. 29, 500036 Brasov, Romania

**Keywords:** deep eutectic solvents, circular economy, lignin valorisation, green extraction process, microwave, lignin valorisation

## Abstract

The current research intended to investigate the suitability of different choline-chloride-based deep eutectic solvents for their role in microwave lignin extraction. Lignin, a widely spread biopolymer in plants and woody structures, is a valuable replacement for fossil-fuel-based materials. While some promising applications have been trialled already, the extraction of this material from its matrix still causes problems. Here, we highlight an efficient and fast method to extract lignin from untreated larch bark with deep eutectic solvents in a standard domestic microwave. We developed a straightforward, green methodology, which can be used on various reaction scales, with materials available to many researchers. Lignin was extracted within only 30 min of microwave irradiation in yields of up to 96%. Compared to traditional deep eutectic extraction by conventional heating, the reaction time was cut by 87% and the energy costs were reduced by 93.5%. The hydrogen bond donors were exchanged and different types, namely acid-based, hydroxyl-based and amide-based donor systems, were evaluated for their suitability concerning microwave lignin extraction. This study presents a novel approach towards energy-efficient and green lignin valorisation, without the inherent need for costly equipment.

## 1. Introduction

Lignin, previously known as an unwanted side product from biorefineries [1], is the second most abundant natural polymer on Earth [2,3,4]. Hence, it would be irresponsible to not investigate its potential for value-added smart materials [5,6,7,8,9,10], especially in the light of the rapid depletion of fossil fuels and the ever-growing danger of climate change. Efficient and green extraction of lignin residues from the agricultural or timber industry can be at the core of valorisation of waste streams. Furthermore, lignin can become a valuable substitute for aromatic chemicals and polymer materials, which are currently produced from fossil fuels [11]. Traditional industrial methodologies used for lignin extraction are the so-called Kraft, soda, sulfite and organosolv treatment processes [12,13]. While these processes do work well enough, they often require very high reaction temperatures of 100–200 °C and long reaction times of several hours [14]. In addition to considerable energy consumption, these parameters also cause multiple unwanted side reactions and altercations in the biopolymer [15]. In order to be considered as a real green alternative, the extraction process for lignin must be accompanied by a minimal amount of waste production and pollution. This cannot be said of current industrial processes, as most of them cause, for example, a considerable wastewater pollution [16]. While the organosolv process has shown great promise in terms of high-purity lignin and a less condensed lignin structure, it comes with its own drawbacks, such as low extraction yields and high pressure and temperature requirements [17]. Recently, deep eutectic solvents (DESs) have been explored as green solvents for biomass extraction due to their favourable chemical and physical properties [18,19,20,21,22]. These solvent systems can be prepared without cumbersome purification by mixing a hydrogen bond acceptor (HBA) with one or more hydrogen bond donors (HBDs) [23,24,25]. Specifically, choline chloride (ChCl) is one of the most frequently used HBAs, as it is not only inexpensive but also biocompatible and biodegradable [26]. Extraction rates of 31% and 52.7% have been reported utilizing, for example, 1-ethyl-3-methylimidazolium acetate [22,27]. Furthermore, the extracted lignin has shown high purity, especially when a carboxylic-acid-based DES was employed [28,29,30]. In the last decade, microwave energy has been successfully employed in organic chemistry to cut down energy requirements, reaction times and solvent requirements [31,32]. This has helped industrial chemistry to make the transition into a greener and more environmentally friendly production [33,34,35]. In comparison to classical heating, microwave irradiation has led to compelling accelerations in reaction times and yields. This is mainly due to the efficient internal heating, generated through the direct coupling of microwave energy with the molecules in the reaction [35]. Another positive effect of this type of irradiation is that it has led to a higher selectivity of the reaction and less unwanted side reactions and bye-products, such as condensation and degradation reactions [11]. The lower residence time of the lignin in the acidic reaction media at high temperatures during microwave extraction reduces these risks and leads to higher-quality lignin [36]. Considering all these advantages, it comes as no surprise that microwave irradiation has also been explored in applications for lignocellulosic materials. As lignin is a highly polar molecule, microwave irradiation can lead to higher extraction yields due to the direct excitation of the polar molecules [37,38,39,40,41,42]. Here, the energy is not converted into heat but is used for direct excitation of the polar molecules through dipole–dipole rotation [38]. This, in turn, can cause certain friction within the woody matrix, resulting in more complete, easier and faster extraction of lignin. In this work we explore the combination of microwave reactions with DES as an efficient and green method to extract lignin from untreated larch bark. We aim to develop a novel, viable, fast and effective exaction method for lignin from larch bark with the possibility for scale up but without the need for costly equipment.

## 2. Materials and Methods

### 2.1. Materials

Fresh bark from larch (*Larix decidua* (Mill.)) trees was provided from Graggaber Gebirgslärche GmbH in Lungau (Austria). Reagents, such as choline chloride, ethanol (99%) and acetic acid were purchased from Carl Roth (Karlsruhe, Germany). Folin–Ciocalteau reagent and hydrochloric acid (32%) were obtained from Merck (Darmstadt, Germany). All chemicals were used as received without further purification.

### 2.2. Bark Preparation

The utilized bark was dried at 60 °C and ground to particles using a cross beater mill (Retsch, Haan, Germany). The fractions below 1000 μm were collected and stored in a regulated environment at 20 °C and 65% relative air humidity upon utilization.

### 2.3. Moisture Content of Larch Bark

First, 12.5% moisture content was determined for the larch bark according to ISO 3130 (1994), which was taken into consideration for the calculation of the yields for the extracted lignin [43]. The optimization reactions were carried out at a 1g scale, resulting in a maximum amount of 0.252 g lignin per reaction for larch bark (28.8% lignin content in larch bark).

### 2.4. Biopolymer Extraction

For all experiments a commercially available domestic microwave Whirlpool M515 was used. The reaction vessel was a 250 mL Schott DURAN borosilicate glass bottle. The DES was prepared by utilizing choline chloride with acetic acid at a molar ratio of 1:2. This mixture was then heated at 200 W for only 2 min prior to the addition of the bark particles. Once the bark had been added, the reaction was heated at 200 W for various lengths of time. All reactions were carried out on a 1 g scale of larch bark and a 1:10 bark:DES weight ratio. After the reaction, the solid and liquid fractions of the reaction mixture were separated via filtration and the solids were washed with ethanol to remove any remaining extracted lignin. The soluble fraction was evaporated and the lignin was then precipitated from H_2_O and the DES was recovered from the aqueous solution after lignin removal. For the large-scale reaction with 10 g a 500 mL Schott DURAN borosilicate glass bottle was used.

### 2.5. Lignin Characterization

ATR–FTIR spectroscopy was performed using a PerkinElmer Frontier FT-IR spectrometer equipped with the ATR MIRacle accessory (PerkinElmer, Waltham, MA, USA). The lignin samples were grinded into a fine powder and applied on the sample tray. For each sample 32 scans with a resolution of 4 cm^−1^ in a wavenumber range between 4000 and 600 cm^−1^ were utilized. The absorbance was measured using P4 UV-Visible Spectrometer from VWR. For this measurement the lignin sample was dissolved in ethanol. The NMR spectra were recorded with an Nanalysis NMReady 60 Pro (60MHz) in *d6*-DMSO.

## 3. Results

### 3.1. Optimization of the DES Preparation

The aim of this work was to provide a novel, viable, fast and effective exaction method for lignin from larch bark, with the possibility for scale up but without the inherent need for costly equipment. Microwave irradiation has revolutionized the organic chemistry sector and has resulted in a dramatic increase in both reaction rate and yield. It is known that this effect can be enhanced in polar solvents, as these have a greater ability to absorb microwave radiation than non-polar solvents. DESs are polar solvents, which have been demonstrated to work well in microwave reactors [44]. However, such reactions often require expensive microwave reactors. We set our goal to develop a reliable and agile methodology that works well even in a standard domestic microwave (MW), making this available and applicable for a broader audience. Schott Duran borosilicate bottles were used as the reaction vessel, as they display a good chemical and pressure resistance. Standard DES preparation involves the mixing and heating of the two to three chemical components for about an hour. In this work, a DES consisting of choline chloride and acetic acid at a molar ratio of 1:2 was utilized. This system was chosen as a valuable starting point as it has been reported to extract lignin efficiently at a very high purity [28,29]. Instead of heating the DES in the conventional way through electric energy, it was decided to not only use the microwave irradiation for the lignin extraction but also for the DES preparation. Hence, choline chloride and acetic acid were added to a reaction vessel and subjected to microwave irradiation at 200 W. After only 2 min, the solution obtained its characteristic clear appearance and was ready to be used for lignin extraction. Compared to previously reported methods, this approach results in an incredible time saving. Due to more efficient heating under microwave irradiation, instead of heating the solvents for 1h to 80 °C, the DES solvent system was available for extraction after only 2 min, shortening the preparation time by 97%.

### 3.2. Optimization of the Reaction Time

**Figure 1:**
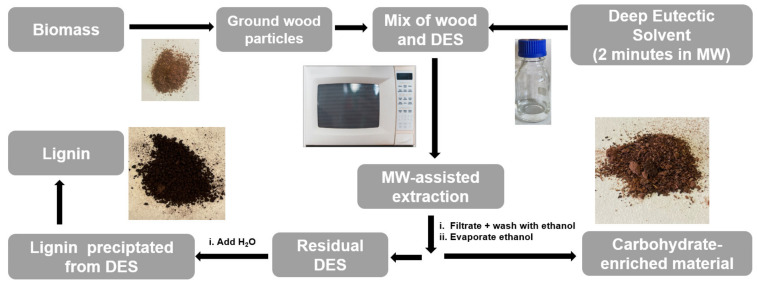
Schematic overview of the microwave–solvothermal extraction process.

The next step involved the optimization of the required reaction time for the optimum extraction rate. A microwave–solvothermal method (see Figure 1) was chosen due to the concomitant rate and yield improvement for microwave reactions, which have been reported synergistically with the benefits of DES as a reaction media [45]. All optimization reactions were performed with 1 g of larch bark. Especially in Europe, larch is used as a construction material, which goes hand in hand with a large larch bark waste stream in the timber industry. Hence, we decided to utilize this former waste material as a source for valuable biopolymers. The bark was directly added to the reaction vessel, which already contained the DES. The reaction was exposed to microwave irradiation for various lengths of time ranging from 2 to 30 min. The amount of extracted lignin was then compared for the different heating durations in order to establish the optimum reaction time for maximum lignin extraction. After only 2 min of irradiation at 200 W, a lignin yield of 24% was obtained (Table 1). Encouraged by this, the reaction time was lengthened to 5 min, at which the yield basically stagnated and stayed around the same percentage. Once the irradiation was further prolonged to 10 min, the extraction yields significantly improved to 53%. While this was already a very satisfying result, further optimizations were undertaken. After 15 min, the yield further improved by 17%, resulting in a total of 70% of lignin being extracted. Another elongation of the reaction time to 20 min resulted in a yield of 89%. While the extension to 25 min did not lead to an increase in the yield, an incredible high extraction rate of 96% was obtained after only 30 min. Prolonged heating as well as higher irradiation than 200 W lead to degradation of the material.

Once the reaction time was optimized, focus was placed on the scalability of this methodology. Therefore, the reaction was scaled up from 1 g larch bark to 10 g of starting material. Thus, 100 g of DES was prepared, as previously mentioned. After 2 min, 10 g of larch bark was added to the reaction vessel and the reaction was heated for 30 min at 200 W. The extracted lignin was separated from the solid residue through washing with ethanol and the solvent was removed and the lignin was precipitated from ethanol/H_2_O. A total amount of 2.28 g of lignin was extracted, which results in an extraction yield of 90%. This is in line with the yields that were accomplished during the optimization process, highlighting that this methodology can easily be upscaled without further adaptations. Once the reaction parameters were optimized, other ChCl-based DESs with other types of HBDs were prepared and tested for their utilization in microwave lignin extraction. Therefore, the previously optimized reaction conditions were applied to the new systems. In addition to another acid-based HBD, a hydroxyl-based HBD as well as an amide-based HBD were tested. These were chosen to evaluate the correlation between the DES solvent system and lignin extraction efficiencies. The HBA stayed the same over the whole process. Two acid-based DESs were generated utilizing acetic acid (AA) and citric acid (CA), one hydroxyl-based DES with diethylene glycol (DG) as well as one amid-based DES with urea (U). All the DESs were again synthesized in the microwave reactor by heating the different components for 2 min at 200 W. Solely for the urea-based DES, heating for 2 min at 400 W was necessary in order to obtain a clear solvent system.

Table 2 summarizes the efficiencies of the different CHCl-based DESs for lignin extraction in the microwave. Acetic acid, which can act as both a hydrogen bond donor and acceptor, afforded the best results. Citric acid and diethylene glycol resulted in nearly the same yields of 75%, while urea gave the lowest amount of lignin with 65%.

### 3.3. Comparison to Traditional DES Extraction and Organosolv Process

In the next step, the microwave methodology for the best working DES (CHCl/AA) was compared to traditional DES extraction by conventional heating, with the same solvent system, as well as another standard extraction process, namely the organosolv process [46,47]. Therefore, for the organosolv process, 10 g of larch bark was placed in a custom-built stainless-steel reactor. Alcohol was added in a ratio of 1:7 (*w*/*w*) as well as 1% (*w*/*w*) concentrated sulfuric acid. The reaction was then performed for 2 h at 160 °C. The extracted lignin was separated from the solid residue through washing with ethanol. The solvent was removed and the lignin was precipitated from ethanol/H_2_O through the addition of hydrochloric acid (HCl). The yield of the ethanol organosolv lignin amounted to 38%. The traditional DES was prepared at the same molar ratio and heated for 1 h at 80 °C. After this time, the larch bark was added to the flask and the temperature was raised to 130 °C. The extraction was allowed to run for 3 h, which resulted in a lignin extraction rate of 93%. While the yield obtained is comparable with the yield achieved through microwave irradiation, the reaction time varies significantly. In order to be able to advertise lignin as a viable and truly green alternative to other polymers, all reaction parameters for its extraction process have to be optimized and the energy and time requirements should be kept at a minimum. This is where the microwave methodology shows its real strengths. While the standard method already requires 1 h of heating at 80 °C for the DES preparation, another 3 h at 130 °C are necessary to achieve similar results to the microwave reaction. The fast and effective heating through microwave irradiation not only reduces the total reaction time from DES preparation to extraction completion by 87%, it also saves a considerable amount of energy. The traditional DES reaction process from DES solvent preparation to the end of the extraction process consumes a total of 2.6 kWh per reaction, based on the power input for the RTC basic heating plate from IKA that was used during the extraction. The MW process only utilizes 0.17 kWh of energy, based on the power input of the microwave. This effectively reduces the active energy costs by 93.5%. Our study proved that high extraction yields of lignin can be achieved with inexpensive equipment at a percentage of the reaction time and energy. Moreover, a variety of DES was trialled and evaluated. When looking for a holistic green extraction method, the utilization of microwave irradiation for extraction processes has clearly proven its superiority and merits.

### 3.4. Spectroscopic Results

**Figure 2:**
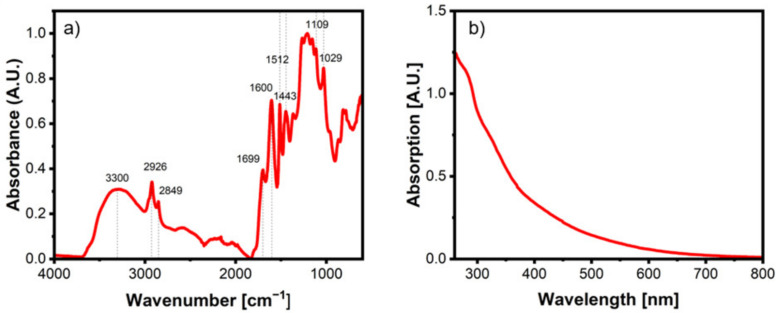
(**a**) FTIR spectra of lignin; (**b**) UV/vis spectrum of lignin.

FTIR spectroscopy, especially, can be a useful tool to determine the chemical functionalities present in the lignin backbone, which, in turn, provides information if the extracted lignin has been condensed and chemically modified during the extraction process. Characteristic lignin bands are the hydroxyl groups in both the phenolic as well as the aliphatic elements of the lignin structure, which appear as a broad band around 3300 cm^−1^ (Figure 2a). Furthermore, the two narrow bands at 2926 and 2849 cm^−1^ resemble the CH stretching of aromatic methoxy groups and methyl or methylene groups, respectively. The aromatic C-C stretching vibrations can be seen in an area of 1600–1443 cm^−1^, with a small band for the carbonyl/carboxy region at 1699 cm^−1^. All bands identified are in alignment with the literature and can be seen in Table 3 [48,49,50,51,52].

**Figure 3:**
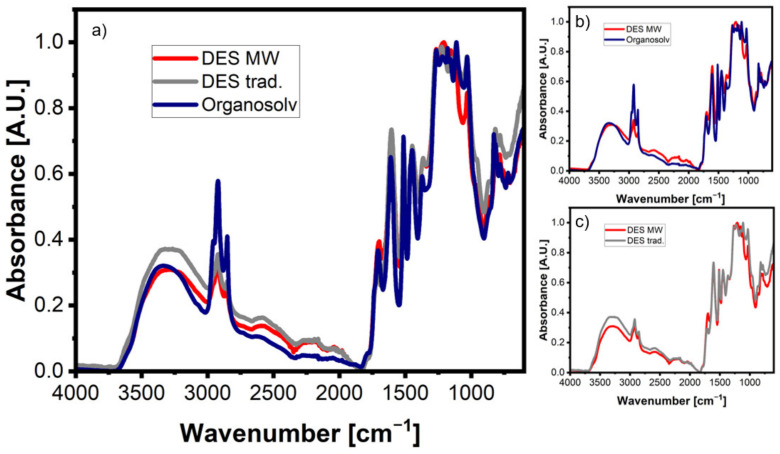
(**a**) FTIR spectra of lignin: MW DES, traditional DES and organosolv; (**b**) comparison of FTIR spectra MW DES and organosolv lignin; (**c**) comparison of FTIR spectra MW DES and traditional DES lignin.

The extracted lignin was also compared to lignin extracted in DES with conventional heating and with ethanol organosolv lignin, in order to ensure that the microwave irradiation did not cause any chemical change in the lignin structure (Figure 3a). At first glance, it can be seen that for all three different methodologies, the extracted lignin contains the main bands associated with lignin. One feature that stands out is that the CH-stretching vibrations around 2926 cm^−1^ are much more distinctive for the organosolv lignin than for the other two DES methods. When the organosolv lignin is separately compared to the microwave DES lignin (Figure 3b) it can be seen that all the other bands are fairly similar, with one exception being the C-C alkyl stretching band at 1029 cm^−1^, which is a bit more pronounced in the organosolv lignin.

The same can be said when the two DES methodologies are compared (Figure 3c). Again, the bands have a good correlation, with the exception being the C-C stretching band, which is a bit more developed in the traditional DES lignin. In addition to FTIR, the microwave lignin was also examined through UV/vis spectroscopy (Figure 2b). The spectrum reveals a broad absorption band in the region of 200–280 nm, stemming from the variety of functional groups being present in the lignin structure [53,54].

**Figure 4:**
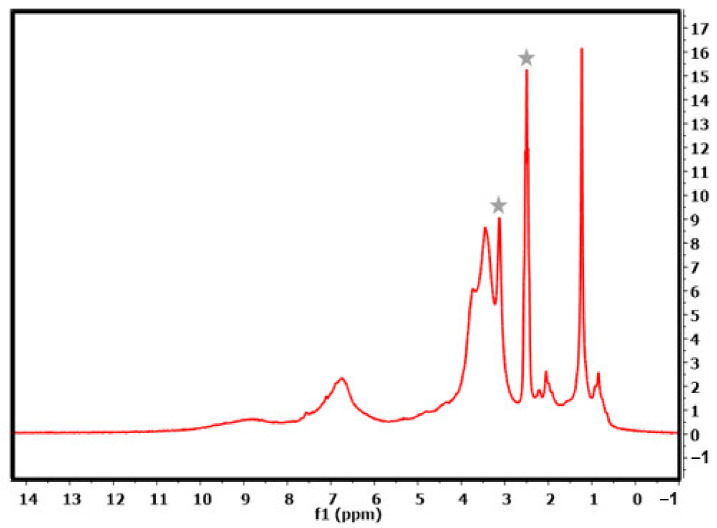
^1^H-NMR of extracted lignin in DMSO-*d6*, solvent and water peaks are marked.

^1^H-NMR spectroscopy represents another way to gain valuable insights and analyse lignin. One example is the extensive study of Lundquist et al. into the phenolic groups of underivatized lignin [55]. DMSO-*d6* was used as solvent, as it is known for its slow proton exchange and, hence, can show the characteristic lignin hydroxyl protons better than other deuterated solvents. The NMR spectrum in Figure 4 shows several clearly resolved peaks, which belong to proton signals of certain structural building blocks of lignin. One example is the spectral range of δ 10.0–9.0 ppm. The signals in this area mainly stem from formyl groups as well as some phenolic hydroxyl groups. Unsubstituted phenolic groups, which are not conjugated to carbonyl groups, can be found in the area of δ 9.0–8.5 ppm. Further up field, between δ 8.5 and 8.0 ppm, signals of substituted phenolic moieties appear. The signal at 7.5–6.0 ppm belongs to the aromatic and vinylic groups in the lignin structure, while the signal 6.0–4.0 ppm stems from aliphatic H_α_ and H_β_ protons. The very prominent broad peak at 4.0–3.3 ppm resembles the methoxy and H_γ_ groups. Further upfield, smaller peaks between 2.2 and 2.0 appear, which belong to hydrogens on aromatic units, while the very sharp peak at 1.5–1.0 ppm belongs to various aliphatic CH_2_-CH_2_ elements. The signal at 0.85 represents the terminal CH_3_ moieties in the lignin structure.

The lignin samples were also analysed concerning their total phenolic content (TPC) with the Folin–Ciocalteu method (Table 4). A high phenolic content of 589 μgGAE/mg, on average, was determined; this was also corroborated by a mean antioxidant inhibition rate of 72%, which normally goes hand in hand with the TPC [56,57,58]. The TPC and the antioxidant activity were then compared to lignin, which was extracted by the traditional DES method through conventional heating. The TPC rate for this type of lignin was also determined to be high, although slightly lower than the microwave lignin, with 522 μgGAE/mg. The antioxidant inhibition was ascertained to be 75% compared to the 72% for the microwave lignin. All in all, it can be said that these values all substantiate our hypothesis that lignin can be extracted utilizing a standard microwave without changing its inherent structure.

## 4. Conclusions

In this study, under the grant of Holz.Aktiv, a fast and effective method to extract lignin from untreated larch bark was developed. By applying microwave heating, the required reaction times were reduced from hours to only minutes and yields were improved in comparison to conventional methods. The maximum extraction yield was obtained after 30 min irradiation, resulting in 96%. The method was also compared to traditional extraction with DES. Here, the preparation time of the DES could be reduced by 97% and the reaction time by 84%. Furthermore, the MW process only utilizes 0.17 kWh of energy compared to 2.6 kWh for the traditional extraction process, reducing the active energy costs by 93.5%. This showcases the inherent benefit in utilizing microwave irradiation for the extraction of biopolymers, both in terms of energy and time efficiency. In conclusion, the combination of deep eutectic solvents with microwave radiation shows great potential for rapid and efficient lignin extraction. Especially with the increasing usage of industrial microwave and continuous flow microwave reactors in industry, our findings can aid the development of further protocols in the future.

## Figures and Tables

**Table 1:** Optimization of the reaction parameter microwave extraction CHCl/AA.

Time (Minutes)	Yield (mg)	Yield (%)
2	60	24
5	55	22
10	134	53
15	177	70
20	225	89
25	201	80
30	243	96

**Table 2:** Comparison of different CHCl-based DESs for microwave extraction.

DES	Yield (mg)	Yield (%)
CHCl/AA	243	96
CHCl/CA	190	75
CHCl/DG	186	74
CHCl/U	165	65

**Table 3:** Assignment of FTIR bands in extracted lignin with DES.

Wavenumber (cm^−^^1^)	Band Assignment
3400–3300	O−H Stretch
2935–2918 and 2859–2850	C–H stretching in methyl and methylene groups and in aromatic methoxyl groups
1712–1686	C=O Stretch in unconjugated ketone, carbonyl and in ester groups (frequently of carbohydrate origin)
1656–1650	C=O stretching in conjugated p-subst. Arylketones
1616–1593	Aromatic skeletal vibrations plus C=O stretching
1518–1505	Aromatic skeletal vibrations
1463–1422	C–H deformations in methyl and methylene and aromatic skeletal vibrations combined with C–H in-plane deformation
1373–1353	Aliphatic C–H stretching in methyl and phenolic OH
1274–1262	G ring breathing plus C=O stretching
1229–1200	C–C plus C–O plus C=O stretching
1168–1150	C=O in ester groups (conjugated) (typical for HGS lignins)
1164–1154	Aromatic C–H in-plane deformation plus secondary alcohols plus C–O stretch
1126–1107	C–O deformation in secondary alcohols and aliphatic ethers
1040–1020	Aromatic C–H in-plane deformation, plus C–O deformation in primary alcohols, plus C=O Stretch (unconjugated)
965–952	–HC=CH-out-of-plane deformations (trans)
879–851/824–802	C–H out-of-plane in positions 2,5, and 6 of G units

**Table 4:** Total phenolic content and antioxidant activity of DES MW lignin.

Total Phenolic Content		Antioxidant Activity		
µg GAE/mg	Abs. (A.U.)	A_15_ (A.U.)	A_0_ (A.U.)	Inhibition (%)
590	2.432	0.155	0.532	71
590	2.431	0.149	0.532	72
588	2.424	0.140	0.532	74

## Data Availability

Not applicable.
